# 173. Antimicrobial Stewardship Education Changes Prescribing Behavior and Reduces Treatment of Asymptomatic Bacteriuria

**DOI:** 10.1093/ofid/ofab466.375

**Published:** 2021-12-04

**Authors:** Niki Arab, Bali Gupta, Brian Kim, Arthur Jeng

**Affiliations:** Olive View-UCLA Medical Center, Sylmar, California

## Abstract

**Background:**

Treatment of asymptomatic bacteriuria (ASB) outside of pregnancy and urological procedures increases the risk of antibiotic resistance without improving outcomes. At Olive View-UCLA Medical Center (Sylmar, CA), the CDC U.S. Antibiotic Awareness Week (AAW) was utilized as a platform to promote antimicrobial stewardship (AS) for ASB. We evaluated the incidence of antibiotic treatment of ASB pre-AAW vs post-AAW, and the impact of AS education on future prescribing practices for ASB.

**Methods:**

In this single-center retrospective observational study, AS education defining ASB vs urinary tract infection (UTI) was provided via visual aids distributed throughout the hospital during AAW from 11/18/2020 to 11/24/2020 (Figure 1). All positive urine cultures (Ucx) for adult inpatients were reviewed prior to AAW from 9/2020 to 11/2020 and after AAW from 12/2020 to 1/2021. Patients were excluded if they were unable to report UTI symptoms, pregnant, or undergoing urological procedure. The incidence of ASB treatment pre- and post-AAW was compared. A survey was sent to providers to compare the impact on antibiotic prescribing behavior for ASB pre- and post-AAW. Fisher’s exact and Chi-squared tests were used for statistical analysis.

Figure 1. Antimicrobial Stewardship Education and Poster Distribution

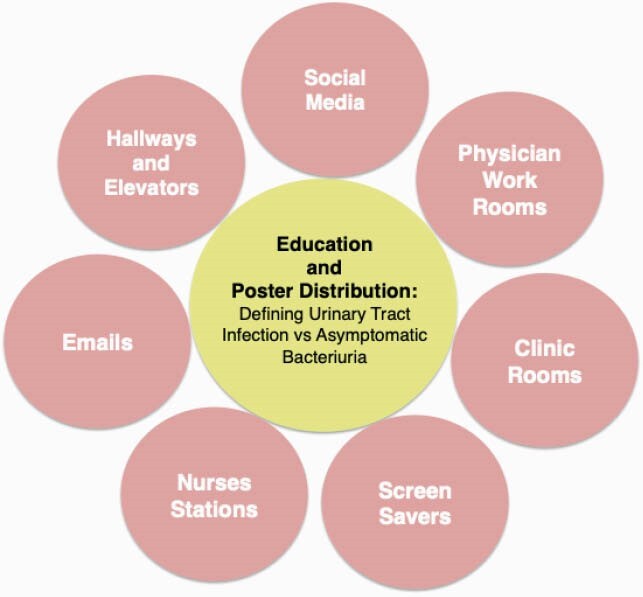

**Results:**

A total of 260 cases met study eligibility. In the pre-AAW group, 56 of 131 cases presented with ASB, of which 16 were treated with antibiotics (28.6%). In the post-AAW group, 55 of 129 cases presented with ASB, and 5 were treated with antibiotics (9.1%). Antibiotics were prescribed more often for patients with ASB in the pre-AAW group compared to those in the post-AAW group (p=0.014). Forty providers completed the survey, of which 97.5% had seen the visual aids, 70% had found the education "very” or “extremely" useful, and 43.6% reported they “always or sometimes” treated ASB pre-AAW vs 15% post-AAW (p< 0.01).

**Conclusion:**

AS posters and education defining ASB significantly decreased the treatment of ASB. AAW education on ASB antimicrobial stewardship demonstrated a high value and shifted prescribing behavior to avoid antibiotic treatment of ASB. A similar approach to deliver provider education could serve as a valuable model to change provider AS practices for ASB.

**Disclosures:**

**All Authors**: No reported disclosures

